# Sexual risk-taking behaviors among young migrant population in Sweden

**DOI:** 10.1186/s12889-022-12996-2

**Published:** 2022-03-30

**Authors:** Sara Causevic, Mariano Salazar, Nicola Orsini, Anna Kågesten, Anna Mia Ekström

**Affiliations:** 1 Department of Global Public Health, Karolinska Institutet, Stockholm, Sweden; 2 Swedish Institute for Global Health Transformation (SIGHT) at the Royal Swedish Academy of Sciences, Stockholm, Sweden

**Keywords:** Migrant, Refugee, Asylum seekers, Sweden, Sexual and reproductive health, Risk behavior, Sexual risk behavior, Adolescents, Youth, HIV

## Abstract

**Background:**

Migration is a complex process of high uncertainty with adjustments to new contexts and experiences influencing individuals’ health. This study aims to assess the prevalence of self-reported sexual risk-taking behaviors among migrant youth population in Sweden, fulfilling the research gap in that field.

**Methods:**

A pre-tested, web-based self-administered cross-sectional survey was used to collect data among 1563 migrant youth (15–25 years old) in Sweden. The survey was conducted in high schools and Swedish language schools for foreigners between December 2018 and November 2019. Pearson chi-square and t-tests were used to compare whether sociodemographic characteristics and migration status varied between those engaging in sexual risk behaviors or not. Multivariate logistic regression was used to determine the adjusted odds ratio of the key outcome variable and independent variables.

**Results:**

There is a profound heterogeneity in migrant youth characteristics related to engagement in different sexual risk-taking behaviors. Those engaging in condomless sex were older, coming from the Americas and Europe, living longer in Sweden and came to live with their family. Belonging to the Islamic religion was a protective factor. Sex under the influence of drugs was related to those from Europe, and Middle East and North Africa (MENA) and coming to Sweden to work/study, where age was a protective factor. Living longer in Sweden, coming for work/study or to live with family had higher odds to engage in sex in exchange for gifts/money.

**Conclusion:**

The results highlight the needed reconsideration of the broader system response that can influence migrant youth health outcomes and public health implications. The approach should consider and relate to sexual risk-taking behavior’s long-term consequences. Migrant youth background needs and knowledge should guide this response.

**Supplementary Information:**

The online version contains supplementary material available at 10.1186/s12889-022-12996-2.

## Introduction

Migration is a complex process of high uncertainty marked with adjustments to a new cultural/social context and experiences that can influence individuals’ health [1]. Approximately 272 million people migrate globally, with 80 million forcibly displaced [2, 3]. Migration is an everyday event with people moving from one place to another due to political, economic, and social factors such as climate change, economic shocks, famine, and political unrest. The perilous journey, the circumstances forcing migration, settling in a new environment, language and cultural differences, limited knowledge and information on the health system, financial and legal status uncertainties in the receiving country, and the need to adapt to new cultural norms, make migrants’ health needs and outcomes complex and sometimes challenging to achieve [1, 4].

### Sexual risk-taking and migration

Sexual risk-taking behavior lacks a clear and unanimously agreed definition [5]. Still, several authors have included, among others, early sexual debut, sexual intercourse without a condom or inconsistent condom use, multiple concurrent and/or sequential partners, transactional sex, and sex under the influence of drugs and alcohol [6–9]. These behaviors can increase the risk of acquiring sexually transmitted infections (including HIV/AIDS), unwanted and teenage pregnancies, and other adverse health outcomes [5]. Sexual risk-taking among youth is a concerning issue because it can negatively affect their health later in life [10].

Studies have shown that sexual risk prevalence can vary among migrant populations, and it could be higher among migrants than non-migrants [11–14]. For example, prevalence can range from 92% in Russia (for intercourse with sexual workers), 68% in Portugal (for condomless last sexual encounter), to five or more lifetime sexual partners than non-migrants in Uganda (25% vs 19%) [11, 12, 14]. Sexual risk-taking behavior among migrants can occur for different reasons, including having sex to fit in and cope with life uncertainties such as family separation, loneliness, and financial hardship [4, 15].

### Migration and Sweden

Immigration is an important part of Swedish society. Since the 1970s, Sweden has experienced several immigration waves with refugees and migrants from Latin America, Africa, Asia, and Europe [16]. Foreign-born citizens constitute 19.7% of the total population of Sweden [17]. The main motives for immigration to Sweden have consistently been war, lack of political freedom in the country of origin, and economic difficulties. More recently, Sweden has had a large influx of migrants in the form of asylum seekers. In 2015 alone, 157,017 people requested asylum, with a significant proportion (43%) being children and adolescents, including unaccompanied minors [3, 18].

Access to sexual and reproductive health in Sweden differs according to migration status. For example, asylum seekers are entitled to “emergency healthcare and dental care, and health care that cannot wait”, a free health assessment to identify any urgent health problems, and free contraception and maternal health care [19].

Refugees are offered an introductory course to the Swedish society where they discuss norms and values about sexuality, reproductive health and sexually transmitted infections (STI) prevention, among other topics [20]. There are no educational information packages available for non-refugee /asylum seeker migrants. Migrant youth attending school in Sweden are exposed to the same sexual education provided to all native-born young people [21].

### Rationale

Achieving good sexual and reproductive health is a key part of the Sustainable Development Goals (SDG), as reflected in SDG 3 (to ensure healthy lives and promote well-being for all at all ages) and SDG 5 (to achieve gender equality and empower all women and girls) [22]. Yet, SDG 3 and 5 will not be achieved if countries do not address sexual and reproductive health determinants and propose remedial action. Tackling poor sexual and reproductive health must be a priority for governments worldwide, especially among migrant youth populations that have shown a higher prevalence of sexual risk-taking than natives and face challenges in accessing sexual and reproductive health services in the host countries, including Sweden [12, 14, 23].

The association between migration and health has been extensively studied in Sweden, providing important evidence to improve the migrants’ sexual and reproductive health. Previous research has consistently shown that people with a migrant background (including second-generation migrants) have poorer physical, mental, and reproductive health than native Swedes [16, 24–26]. However, very few quantitative studies have assessed the role of migration in sexual risk-taking behaviors among migrant youth in Sweden.

We will contribute to filling this knowledge gap by mapping the prevalence of selected self-reported sexual risk-taking behaviors and assessing which migration-related factors (i.e., the main reason to come to Sweden, having a residence permit, etc.) influence migrant youth populations’ sexual risk-taking. Understanding sexual risk-taking behavior is vital to improve migrant youth sexual health and curtail the risk of acquiring a STI, including HIV, in the post-migratory phase [12].

## Materials and Methods

### Study design and population

In our paper, we use the International Organization for Migration (IOM) definition of a migrant, defined as “*a person who moves away from his or her place of usual residence, whether within a country or across an international border, temporarily or permanently, and for a variety of reasons*” [27]. In this study, we focus on international migration to Sweden. Our target population were migrants 15–25 years old.

**Fig. 1:**
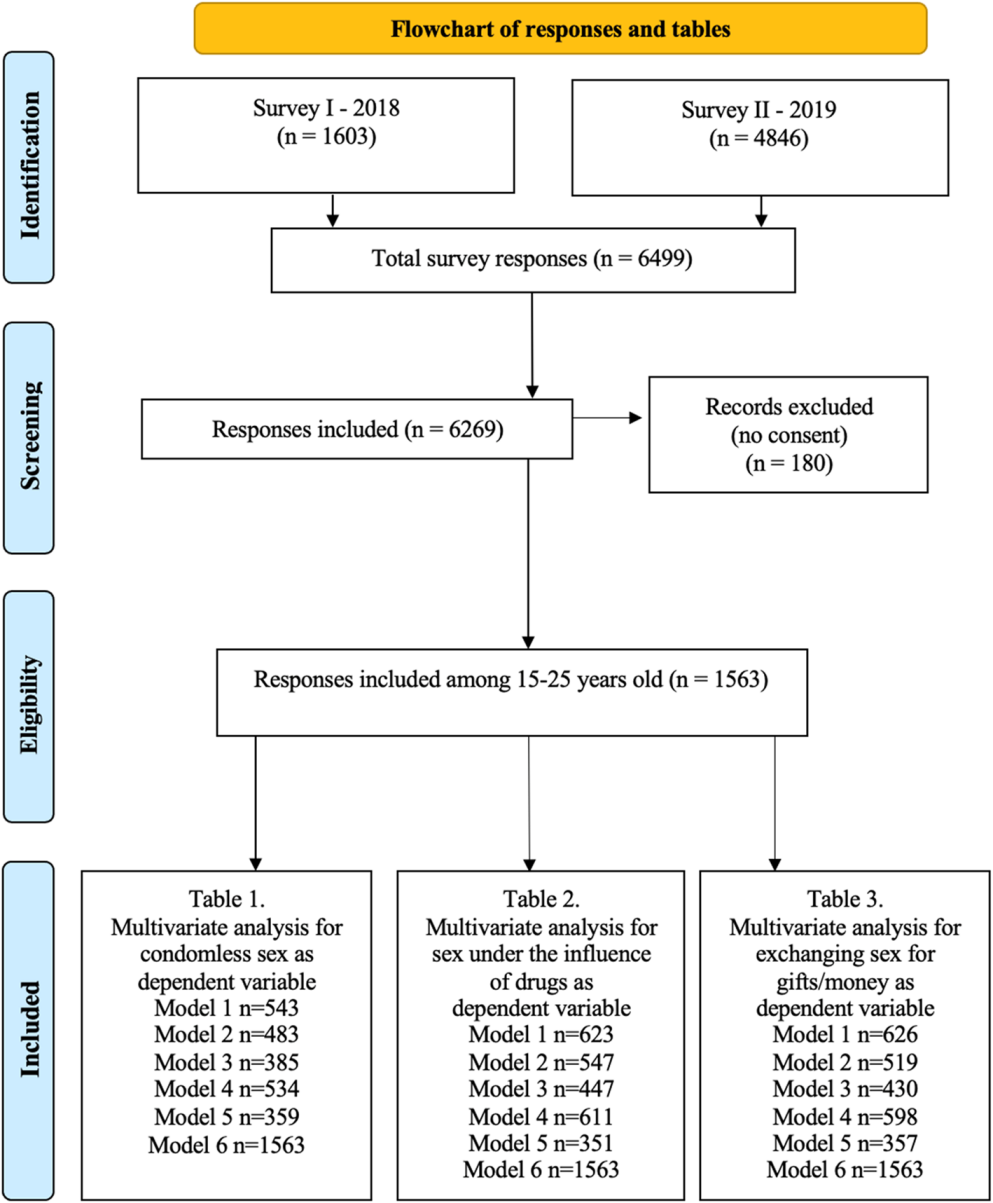
Flowchart of the number of participants, responses, and tables

To collect the data, we conducted a cross-sectional study in Sweden (December 2018–November 2019). This study was part of a larger project aimed to study sexual and reproductive health and rights, knowledge, attitudes, practices, outcomes, and the health system response to migrants’ needs in Sweden [28]. In total, the data was collected from 6269 people, with 1563 aged between 15–25 years and thus eligible for our study (Fig. 1).

### Data collection

Data was collected among migrants in institutions offering Swedish language for foreigners (Svenska För Invandrare (SFI)) or Swedish as a second language (SVenska som Andra sprak (SVA)) and at upper elementary schools offering education to recently arrived migrant youth. Trained research assistants visited the classrooms in each school and all students were invited to participate.

Data was collected using a self-completion online survey piloted before implementation. Students who agreed were provided with a link and password to the survey website. The survey was available in Arabic, English, Farsi, Tigrinya, Spanish, Swedish, and Somali. For this study, 44.42% reported attending high school, 36.61% attended SFI school, and 18.97% attended SVA school.

### Study variables

Variables that have been shown in previous studies to influence main outcome variables (sex, age, education, religion, country born and raised, and the number of years living in Sweden) and migration-related variables of interest (the main reason to come to Sweden, having a residence permit, and current living arrangements) were included in the analyses [5, 24, 29, 30].

#### Main outcome variables

The main outcome variables were in the domain of sexual risk-taking behavior in the past year:

- In the last year, have you had sex without a condom (*Yes* or *No* or *Never had sex*),
- In the last year, have you been under the influence of drugs during sex with a casual partner? (*Yes* or *No* or *Never had sex*),
- In the last year, have you had sex in exchange for gifts/money (*Yes* or *No* or *Never had sex*).

For each of the variables, we created a dichotomous variable (*Yes, No*). Those who have answered *Yes* were coded as 1, and *No* as 0. Those who answered “never had sex” to any of the questions described above were excluded from the analysis.

#### Migration-related variables

Three variables were measured: 1. The main reason to come to Sweden (*I came as an Asylum seeker /refugee*, or *I came to work/study*, or I *came to live with family,* or *Other*), 2. Having a residence permit (*No*, or *Yes*, or *I am an EU/EEA/Swedish resident*), and 3. Current living arrangements (*Alone*, or *Married or cohabiting*, or *With other family*, or *With friends I knew*, or *In a refugee home*).

#### Demographic variables

Sociodemographic variables measured were: 1. Sex (*Male,* or *Female,* or *I don’t want to answer),* 2. Ag*e (years),* 3*.* Education *(years),* 4. The number of years living in Sweden *(years),* 5. Religion *(Christianity, Islam, Other religion, no religion) and* 6. Country born or raised.

The country variable was grouped into eight regions: *1) Africa, 2) Americas, 3) Asia, 4) Australia, Other, Stateless, 5) Europe, 6) Syria, 7) Afghanistan, and 8) the Middle East, North Africa (MENA) countrie*s, without Syria (Algeria, Bahrain, Egypt, Iran, Iraq, Israel, Jordan, Kuwait, Lebanon, Libya, Morocco, Oman, Qatar, Saudi Arabia, Palestine, Tunisia, United Arab Emirates, Yemen). Syria and Afghanistan were separated due to most of the migrants coming to Europe from these two countries at the time of conceptualizing this study [31].

### Data analysis

We used Pearson chi-square and t-tests to compare if sociodemographic and migration-related characteristics varied between groups. Multivariable logistic regression models were used to estimate adjusted odds ratios (AOR) and 95% confidence intervals (CIs). All analyses were considered significant if *P* < 0.05. We evaluated the patterns of missing data before engaging in multivariable analysis. Demographic and migration-related variables were 72 and 58% complete, respectively. The percent of missing data was 16% for religion.

Performing just a complete case analysis would have made us discard incomplete records that contain useful information. Model 1 included sociodemographic variables. Then, we fitted separate models for each migration-related variable (Models 2–4) followed by a complete-case analysis (Model 5). All models were adjusted for the sample demographic characteristics.

We imputed the data using a multiple imputation model by chained equations (MICE) and combined the estimates using Rubin’s rule [32]. This was an attempt to include all participants and reduce bias and increase precision. Missing data could reduce the power of analysis and increase bias. Finally, as suggested by reviewers, we performed a sensitivity analysis by removing migrant youth who reported being single form the multivariable models (see Supplementary material, Additional file 2). Data were analyzed using Stata v16 (StataCorp, College Station, Texas).

## Results

### Demographic characteristics

Participants’ mean age of 19.65 years (SD = 2.7) of which 55.8% were men. Almost half of the participants (41.5%) had a high school or higher education. The mean number of years living in Sweden was 2.5 (SD = 1.51). Most belonged to the Islamic religion (61.1%). The most common regions of origin were Africa and Syria (22% each). 61,2% came to Sweden as asylum seekers or refugees and 86.8% had a residence permit. Most were currently living with their immediate family members (54.9%) (Additional file 1).

### Sexual risk-taking prevalence and associated factors, univariable analysis

**Fig. 2:**
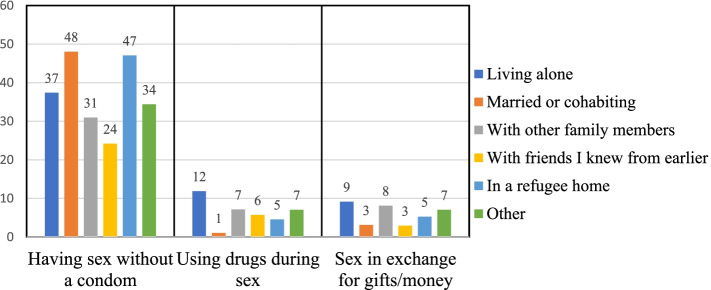
Illustration of sexual risk-taking behaviors and current living situation prevalence, univariable analysis

The prevalence estimate for the participants who reported having sex without a condom in the last year was highest among those married or living with a partner (48%), followed by those living alone (37%) (Fig. 2). Among those who had used drugs during sex in the last year, the highest prevalence was among those who lived alone (12%) or with family members (7%), whereas those who were married had the lowest prevalence (1%) (Fig. 2). When exchanging sex for drugs or money, the prevalence was highest for those living alone (9%) and those living with other family (8%) but the relationship was not significant (Fig. 2).

Univariable analysis showed that having sex without a condom was associated (*p* < 0.05) with religion, country born and raised, and current living arrangements (see Supplementary material, Additional file 1). The bivariate analysis also showed that using drugs during sex in the last twelve months was associated (*p* < 0.05) with being male, country born/raised, the reason to come to Sweden, living arrangements, and residence status. Finally, exchanging sex for money or gifts was associated (*p* < 0.05) with reasons to come to Sweden (Additional file 1).

### Multivariable analysis

In the following, we show the estimates of the adjusted ORs and 85% CI obtained with a multivariable logistic regression model for each of our three outcomes.

Our first outcome variable was condomless sex in the last year. Our multivariable analysis showed that after adjusting for other covariables, increasing age (Table 1, Models 1–5), increasing years of stay in Sweden (Table 1, Models 1–5), born in the American continents (Table 1, Models 1,2 and 4) and Europe (Table 1, Model 1 and 4), and coming to Sweden to live with family (Table 1, Model 2) increased the odds of having sex without a condom in the last year. Reporting Islam as one’s religion (Table 1, Model 5) was a protective factor against condomless sex in the last year.

**Table 1:** Association between condomless sex in the last year, demographic characteristics and migration-related variables, adjusted odds ratios (AOR) and 95% confidence intervals (CI) shown

Characteristics	Model 1 (***n*** = 543)	Model 2 (***n*** = 483)	Model 3 (***n*** = 385)	Model 4 (***n*** = 534)	Model 5 (***n*** = 359)	Model 6 (***n*** = 1563)
	AOR (95%CI)	AOR 95% CI	AOR 95% CI	AOR 95%CI	AOR 95%CI	AOR 95%CI
**Sex (ref: Male)**	1.00 (reference)					
Female	0.83 (0.55–1.25)	0.63 (0.39–1.03)	0.86 (0.51–1.45)	0.77 (0.49–1.22)	0.72 (0.39–1.33)	0.80 (0.51–1.27)
*Age (years)	1.00 (reference)					
*Mean and SD	1.11 (1.03–1.19)**	1.13 (1.04–1.23) **	1.14 (1.04–1.25)**	1.10 (1.01–1.19)*	1.14 (1.01–1.27)*	1.09 (0.98–1.21)
**Education (years)** **(ref: less than 3 years)**	1.00 (reference)					
4–6	0.78 (0.43–1.40)	0.72 (0.39–1.31)	0.92 (0.46–1.79)	0.80 (0.44–1.48)	0.90 (0.44–1.81)	0.68 (0.37–1.25)
7–9	0.82 (0.42–1.62)	0.80 (0.37–1.74)	0.96 (0.36–2.52)	0.83 (0.41–1.68)	1.32 (0.45–3.84)	0.63 (0.35–1.14)
10 or longer	0.95 (0.51–1.76)	0.79 (0.41–1.55)	1.10 (0.53–2.29)	1.05 (0.55–2.00)	1.16 (0.52–2.58)	0.90 (0.44–1.84)
*Religion (ref: other religion, atheist)	1.00 (reference)					
Christianity	1.14 (0.62–2.09)	1.20 (0.61–2.38)	1.36 (0.62–2.99)	1.18 (0.62–2.20)	1.09 (0.47–2.51)	1.15 (0.64–2.10)
*Islam	0.68 (0.40–1.14)	0.66 (0.36–1.19)	0.73 (0.37–1.44)	0.61 (0.36–1.05)	0.47 (0.22–0.97)*	0.69 (0.35–1.32)
*Living in Sweden (years) Mean and SD	1.17 (1.00–1.35) *	1.24 (1.04–1.48) *	1.20 (1.00–1.44)*	1.26 (1.08–1.48)**	1.41(1.14–1.74)**	1.29 (1.09–1.52)**
*Country/Born and raised (ref: Syria)	1.00 (reference)					
Other, Australia, Asia	1.13 (0.41–3.12)	0.41 (0.09–1.72)	0.82 (0.20–3.17)	1.22 (0.43–3.40)	0.37 (0.07–1.90)	1.06 (0.33–3.36)
*Americas	2.90 (1.49–5.64)**	2.15 (1.05–4.41)*	1.90 (0.91–3.95)	3.05 (1.54–6.00)**	1.60 (0.73–3.50)	2.31 (1.07–4.98)*
*Europe	2.28 (1.08–4.77)*	1.65 (0.67–4.00)	2.03 (0.65–6.34)	2.61 (1.19–5.68)*	1.36 (0.35–5.16)	3.09 (1.19–8.01)*
Africa	0.76 (0.38–1.50)	0.72 (0.34–1.47)	0.74 (0.32–1.66)	0.79 (0.38–1.61)	0.62 (0.26–1.48)	0.77 (0.40–1.50)
MENA	1.69 (0.88–3.22)	1.58 (0.76–3.26)	1.22 (0.56–2.64)	1.67 (0.85–3.29)	0.91 (0.37–2.23)	1.57 (0.74–3.32)
Afghanistan	0.94 (0.48–1.83)	0.87 (0.43–1.73)	0.68 (0.31–1.49)	0.89 (0.43–1.82)	0.54 (0.22–1.31)	0.95 (0.44–2.06)
*Main reason to come to Sweden **(ref: As an asylum seeker/refugee)**	1.00 (reference)					
To work/study		1.96 (0.94–4.06)			2.44 (0.87–6.80)	1.55 (0.75–3.19)
*To live with family		2.33 (1.23–4.38)**			1.99 (0.95–4.13)	1.38 (0.82–2.31)
Other		1.37 (0.52–3.60)			2.15 (0.72–6.46)	1.40 (0.47–4.10)
**Having a residence permit (ref: EU/EEA/Swedish citizen)**	1.00 (reference)					
No			3.20 (0.69–14.69)		3.31 (0.56–19.55)	1.66 (0.42–6.55)
Yes			3.52 (0.83–14.87)		3.99 (0.72–22.29)	1.98 (0.65–6.06)
**Current living arrangements** **(ref: with other family)**	1.00 (reference)					
Alone				1.05 (0.61–1.79)	1.22 (0.59–2.50)	1.23 (0.81–1.89)
Married or cohabiting				1.63 (0.83–3.19)	1.38 (0.59–3.20)	1.69 (0.95–2.99)
With friends I knew from earlier				0.52 (0.25–1.08)	0.76 (0.32–1.78)	0.52 (0.22–1.23)
In a refugee home				2.51 (0.78–8.03)	1.38 (0.26–7.14)	2.01 (0.55–7.28)

Legend: * *p* < 0.05; ** *p* < 0.01

*MENA countries (Algeria, Bahrain, Egypt, Iran, Iraq, Israel, Jordan, Kuwait, Lebanon, Libya, Morocco, Oman, Qatar, Saudi Arabia, Palestine, Syria (excluded), Tunisia, United Arab Emirates, Yemen)

Crude OR for considered explanatory factors. Model 1, Adj. OR for sociodemographic factors: sex, age, country born or raised, religion, number of years living in Sweden, education, Model 2, Adj., OR factors included in Model 1 + Main reason to come to Sweden; Model 3 included Model 1+ Having a residence permit in Sweden; Model 4 included Model 1 + Current living arrangements. Model 5 included all variables. Model 6 is a multiple imputation model

Our second outcome was having sex under the influence of drugs during the last year. The multivariable analysis showed that after adjusting for other covariables, the odds of using drugs while having sex during the previous year increased for those reporting being born in Europe (Table 2, Models 1,2,4), being born in a MENA country (Table 2, Models 1–4), and coming to Sweden to work or study (Table 2, Model 2). Being younger in age was a protective factor against using drugs while having sex (Table 2, Model 2).

**Table 2:** Association between having sex under the influence of drugs in the last year, demographic characteristics and migration-related variables, adjusted odds ratios (AOR) and 95% confidence intervals (CI) shown

Characteristics	Model 1 (***n*** = 623)	Model 2 (***n*** = 547)	Model 3 (***n*** = 447)	Model 4 (***n*** = 611)	Model 5 (***n*** = 351)	Model 6 (***n*** = 1563)
	AOR (95%CI)	AOR 95% CI	AOR 95% CI	AOR 95%CI	AOR 95%CI	AOR 95%CI
**Sex (ref: Male)**	1.00 (reference)					
Female	0.50 (0.23–1.07)	0.38 (0.15–1.00)	0.36 (0.12–1.05)	0.61 (0.27–1.35)	0.31 (0.07–1.27)	0.70 (0.33–1.50)
*Age (years)	1.00 (reference)					
Mean and SD	0.87 (0.75–1.01)	0.83 (0.69–0.98)*	0.87 (0.71–1.05)	0.93 (0.78–1.09)	0.86 (0.66–1.12)	0.88 (0.72–1.09)
**Education (years)** **(ref: less than 3 years)**	1.00 (reference)					
4–6	1.42 (0.52–3.87)	1.64 (0.56–4.79)	1.73 (0.54–5.49)	1.40 (0.48–4.07)	1.67 (0.47–5.91)	1.34 (0.42–4.27)
7–9	2.06 (0.69–6.11)	1.77 (0.49–6.26)	2.07 (0.47–9.14)	2.32 (0.73–7.30)	2.45 (0.47–12.56)	1.45 (0.33–4.54)
10 or longer	1.57 (0.51–4.85)	1.93 (0.56–6.61)	2.19 (0.58–8.24)	1.81 (0.55–5.88)	2.38 (0.54–10.58)	1.72 (0.52–5.69)
**Religion (ref: other religion, atheist)**	1.00 (reference)					
Christianity	1.31 (0.49–3.47)	0.97 (0.32–2.90)	0.85 (0.21–3.38)	1.40 (0.50–3.85)	0.72 (0.16–3.19)	1.04 (0.32–3.40)
Islam	0.67 (0.29–1.51)	0.68 (0.27–1.68)	0.80 (0.28–2.20)	0.62 (0.27–1.42)	0.54 (0.17–1.68)	0.75 (0.31–1.79)
**Living in Sweden (years)** Mean and SD	1.25 (0.94–1.67)	1.37 (0.98–1.92)	1.00 (0.71–1.41)	1.24 (0.92–1.67)	1.11 (0.72–1.69)	1.31 (0.92–1.87)
*Country/Born and raised (ref: Syria)	1.00 (reference)					
Other, Australia, Asia	4.43 (0.79–24.60)	1.05 (0.08–12.62)	1.99 (0.23–17.43)	4.36 (0.77–24.68)	0.39 (0.02–7.08)	1.43 (0.11–18.31)
Americas	3.72 (0.82–16.74)	2.31 (0.45–11.73)	1.51 (0.26–8.79)	3.41 (0.74–15.73)	1.15 (0.17–7.75)	1.98 (0.25–16.10)
*Europe	8.81(2.02–38.37)**	7.47 (1.46–38.34)*	2.37 (0.31–17.77)	7.44 (1.64–33.58)**	0.94 (0.07–12.18)	4.75 (0.98–22.89)
Africa	1.34 (0.27–6.58)	1.66 (0.33–8.35)	1.53 (0.27–8.68)	1.14 (0.22–5.83)	1.32 (0.21–8.22)	1.54 (0.22–10.77)
*MENA	5.95 (1.51–23.37)*	5.15 (1.17–22.69)*	5.60 (1.26–24.89)*	6.68 (1.65–26.87)**	4.31 (0.78–23.72)	4.61 (0.87–24.46)
Afghanistan	3.61 (0.89–14.62)	3.65 (0.86–15.51)	3.05 (0.68–13.65)	3.76 (0.87–16.26)	2.22 (0.43–11.55)	2.83 (0.66–12.10)
*Main reason to come to Sweden **(ref: As an asylum seeker/refugee)**	1.00 (reference)					
*To work/study		5.00 (1.69–14.75)**			3.28 (0.71–15.22)	2.89 (0.92–9.05)
To live with family		1.61 (0.38–6.81)			2.29 (0.46–11.48)	1.21 (0.21–6.92)
Other		0.57 (0.07–4.87)			0.83 (0.09–7.68)	0.94 (0.22–4.06)
**Having a residence permit** **(ref:EU/EEA/Swedish citizen)**	1.00 (reference)					
No			2.88 (0.23–37.03)		1.49 (0.08–27.43)	2.55 (0.26–24.78)
Yes			0.82 (0.06–10.05)		0.57 (0.03–10.12)	0.87 (0.14–5.52)
**Current living arrangements** **(ref: with other family)**	1.00 (reference)					
Alone				1.21 (0.50–2.89)	1.85 (0.52–6.56)	1.80 (0.54–5.89)
Married or cohabiting				0.21 (0.02–1.80)	(empty)	0.28 (0.02–3.89)
With friends I knew from earlier				0.62 (0.18–2.03)	1.23 (0.28–5.24)	0.58 (0.10–3.33)
In a refugee home				0.51 (0.05–4.65)	1.25 (0.09–17.38)	0.38 (0.04–3.32)

Legend: * *p* < 0.05; ** *p* < 0.01

*MENA countries (Algeria, Bahrain, Egypt, Iran, Iraq, Israel, Jordan, Kuwait, Lebanon, Libya, Morocco, Oman, Qatar, Saudi Arabia, Palestine, Syria (excluded), Tunisia, United Arab Emirates, Yemen)

Crude OR for considered explanatory factors. Model 1, Adj. OR for sociodemographic factors: sex, age, country born or raised, religion, number of years living in Sweden, education. Model 2, Adj., OR factors included in Model 1 + Main reason to come to Sweden; Model 3 included Model 1+ Having a residence permit in Sweden; Model 4 included Model 1 + Current living arrangements. Model 5 included all variables. Model 6 is a multiple imputation model

Our third outcome was having exchanged sex for money or gifts in the last year. The multivariable analysis showed that after adjusting for other covariables, the increase in years of living in Sweden (Table 3, Model 5) coming to Sweden to work/study (Table 3, Model 2) and to live with family (Table 3, Model 5), and not having a residence permit (Table 3, Model 5) increased the odds of having exchanged sex for money/gifts in the last year.

**Table 3:** Association between having exchanged sex for gifts/money in the last year, demographic characteristics and migration-related variables, adjusted odds ratios (AOR) and 95% confidence intervals (CI) shown

Characteristics	Model 1 (***n*** = 626)	Model 2 (***n*** = 519)	Model 3 (***n*** = 430)	Model 4 (***n*** = 598)	Model 5 (***n*** = 357)	Multiple imputations (***n*** = 1563)
	AOR (95%CI)	AOR 95% CI	AOR 95% CI	AOR 95%CI	AOR 95%CI	AOR 95%CI
**Sex (ref: Male)**	1.00 (reference)					
Female	0.80 (0.38–1.66)	0.64 (0.27–1.52)	0.78 (0.30–1.99)	0.90 (0.41–1.95)	0.76 (0.23–2.48)	0.90 (0.48–1.68)
**Age (years)**	1.00 (reference)					
Mean and SD	0.95 (0.83–1.09)	0.95 (0.81–1.11)	1.04 (0.88–1.23)	0.97 (0.83–1.12)	1.10 (0.89–1.35)	0.99 (0.82–1.19)
**Education (years)** **(ref: less than 3 years)**	1.00 (reference)					
4–6	1.39 (0.49–3.91)	1.50 (0.49–4.52)	1.99 (0.63–6.35)	1.87 (0.59–5.91)	2.14 (0.58–7.83)	1.00 (0.25–3.99)
7–9	1.02 (0.29–3.67)	0.54 (0.09–3.09)	0.56 (0.06–5.49)	1.44 (0.35–5.77)	0.98 (0.08–11.65)	0.54 (0.11–2.57)
10 or longer	1.51 (0.48–4.71)	1.82 (0.53–6.23)	2.28 (0.58–8.87)	2.38 (0.68–8.28)	3.76 (0.82–17.09)	1.06 (0.18–6.21)
**Religion (ref: other religion, atheist)**	1.00 (reference)					
Christianity	1.97 (0.69–5.57)	1.48 (0.46–4.70)	0.71 (0.16–3.12)	2.45 (0.79–7.55)	0.42 (0.08–2.21)	1.45 (0.42–5.00)
Islam	0.86 (0.34–2.19)	0.80 (0.27–2.31)	0.66 (0.21–2.06)	0.91 (0.33–2.48)	0.31 (0.08–1.19)	0.99 (0.39–2.56)
*Living in Sweden (years) Mean and SD	1.22 (0.92–1.61)	1.33 (0.96–1.84)	1.24 (0.89–1.71)	1.32 (0.98–1.76)	1.63 (1.05–2.51)*	1.21 (0.98–1.50)
**Country/Born and raised (ref: Syria)**	1.00 (reference)					
Other, Australia, Asia	3.38 (0.69–16.49)	0.83 (0.08–8.98)	2.12 (0.30–14.75)	3.67 (0.73–18.37)	0.25 (0.01–3.87)	1.91 (0.40–9.14)
Americas	1.62 (0.41–6.50)	1.00 (0.22–4.63)	1.26 (0.27–5.85)	1.63 (0.39–6.69)	0.70 (0.12–3.88)	1.45 (0.40–5.23)
Europe	3.65 (0.97–13.80)	2.47 (0.53–11.41)	1.43 (0.12–15.81)	3.71 (0.91–15.07)	(empty)	2.55 (0.53–12.21)
Africa	1.33 (0.37–4.80)	1.75 (0.46–6.59)	2.32 (0.58–9.29)	1.31 (0.34–4.98)	2.24 (0.48–10.26)	1.49 (0.29–7.63)
MENA	1.99 (0.53–7.38)	1.47 (0.31–6.95)	1.26 (0.26–5.88)	2.55 (0.66–9.73)	0.79 (0.11–5.55)	1.24 (0.20–7.58)
Afghanistan	1.91 (0.54–6.75)	2.12 (0.57–7.89)	2.08 (0.52–8.29)	2.50 (0.65–9.51)	2.05 (0.42–10.03)	1.70 (0.44–6.61)
*Main reason to come to Sweden **(ref: As asylum seeker/refugee)**	1.00 (reference)					
*To work/study		4.62 (1.47–14.53)**			4.93 (0.82–29.50)	2.36 (0.95–5.87)
*To live with family		2.55 (0.80–8.12)			4.14 (1.03–16.51)*	1.39 (0.53–3.62)
Other		(empty)			(empty)	0.67 (0.06–7.49)
**Having a residence permit** **(ref: I am an EU/EEA/Swedish citizen)**	1.00 (reference)					
No			2.08 (0.58–7.36)		3.20 (0.61–16.67)	1.27 (0.08–18.80)
Yes			(omitted)		(omitted)	0.83 (0.05–12.68)
**Current living arrangements** **(ref: with other family)**	1.00 (reference)					
Alone				0.91 (0.36–2.28)	1.95 (0.58–6.49)	1.02 (0.42–2.47)
Married or cohabiting				0.47 (0.12–1.87)	0.28 (0.03–2.66)	0.43 (0.12–1.55)
With friends I knew from earlier				0.31 (0.07–1.44)	0.54 (0.08–3.51)	0.33 (0.05–1.96)
In a refugee home				(empty)	(empty)	0.60 (0.04–9.49)

Legend: * *p* < 0.05; ** *p* < 0.01

*MENA countries (Algeria, Bahrain, Egypt, Iran, Iraq, Israel, Jordan, Kuwait, Lebanon, Libya, Morocco, Oman, Qatar, Saudi Arabia, Palestine, Syria (excluded), Tunisia, United Arab Emirates, Yemen)

Crude OR for considered explanatory factors. Model 1, Adj. OR for sociodemographic factors: sex, age, country born or raised, religion, number of years living in Sweden, education. Model 2, Adj., OR factors included in Model 1 + Main reason to come to Sweden; Model 3 included Model 1+ Having a residence permit in Sweden; Model 4 included Model 1 + Current living arrangements. Model 5 included all variables. Model 6 is multiple imputations

In our multiple imputation models (Tables 1, 2, 3, Model 6), we found that all variables significant in the previous models showed the same direction of association (risk or protective factor) and similar magnitude (AOR) and confidence intervals. Finally, we performed all analysis excluding 

those who reported currently living with a partner. Very few differences were found.

## Discussion

Our findings show that the prevalence of specific sexual risk behaviors in the last year varied according to whom the respondent was living with in Sweden. The prevalence of exchanging sex for drugs or gifts was highest for those living alone (9%) and those living with other family members (not partner) (8%). In addition, the prevalence of having sex without a condom was highest among those married or living with a partner (48%), followed by those living in a refugee home (47%) and those living alone (37%). Those living alone or with other family members (not partner) had a higher prevalence of using drugs during sex in the last twelve months (12 and 7%, respectively). Risk and protective factors for the behaviors described above varied depending on the type of sexual risk-taking assessed with no consistent pattern across outcomes. In the following, we will discuss our findings by each study outcome.

The high prevalence of condomless sex among those who were married can be explained by a high level of trust among partners living in a monogamous relationship and the possible use of long term contraceptive methods such as intrauterine devices or injectables methods. In addition, the high prevalence of condomless sex among those living in refugee homes should be assessed carefully since the number of people in this group was small (*n* = 17)). Lastly, access to condoms among those living in refugee homes could be a challenge.

Although it is difficult to directly compare our prevalence with other studies due to different time frames (e.g., last 12 months vs last sexual intercourse) or study populations measured, the prevalence of condomless sex among those living alone or with family members (37%) other than partners (30%) found in our study is within the range reported in other studies where almost a third of migrant youth did not use condoms in their last sexual intercourse [26, 33, 34]. In addition, the sexual risk-taking prevalence among migrant youth in our study is similar to the results reported by Asamoah et al. (2018) and The Public Health Agency of Sweden (2020), where they found that 30–32% of migrant youth didn’t use condoms in their last sexual intercourse [26, 33]. Another national study by The Public Health Agency of Sweden in 2017 (also known as UngKAB15 - a survey on Knowledge, Attitudes and Behavior) on sexuality and health among 7755 young people 16–29 old in Sweden (with 10% representing foreign-born individuals) reported a total of 25% of participants used condoms during their last intercourse and 11% have had chlamydia [35]. Further research shows that unprotected sex was more frequent among immigrant youth men than in young men with a Swedish background, revealing that being a migrant could be a risk factor for sexual risk-taking [36]. Contrary to these findings, studies among young people in Sweden found no significant differences in sexual risk-taking between native Swedes and second-generation youth or show different prevalence among migrants of all ages [34, 35, 37–40]. Even though there are still contradictions on this issue worth exploring further, both in Sweden and abroad, we reason that our findings are an important alert to policymakers, service providers, and the broader community.

After adjusting for other demographic and migration-related variables, being older increased the odds of having sex without a condom in the last year. This is in line with findings reported among the migrant population in Portugal and Kenya [37, 41]. One possible explanation for this finding is that as age increases, youth are more likely to have several sexual partners thus, increasing the odds of condomless sex.

Compared to being born in Syria, being born in the American continents and Europe was a risk factor for condomless sex. This finding is similar to findings from a study where postmigration HIV acquisition was higher for Latin America and Caribbean migrants than for other migrants arriving from Sub-Saharan Africa arriving in Europe [30]. This finding could be explained by cultural differences where migrant youth coming from the American continent [42] or Europe have a more open and tolerant attitude to sex and sexuality in general than those coming from Syria.

Our findings indicate that the number of years spent living in Sweden is a factor for engaging in condomless behavior even after adjusting for age and other demographic factors. This is in line with results from other studies showing that the length of stay in the host country could change migrant youth risk perception contributing to increased risk behavior [30, 43]. A possible pathway explaining our result is that engaging in sexual risk-taking behavior can be a coping mechanism of adaptation and desire for assimilation to a new environment with different cultural practices around sex [26, 41, 44]. As migrant youth from countries with more restrictive views on sexuality learn how to navigate the more open sexual landscape in Sweden, they might engage in risky sexual behavior as part of this learning process [16, 45]. In addition, the prevalence of sexual partners and condomless sex is increasing in Sweden, especially among the younger population, so the peer pressure to engage in condomless sex could also explain our findings [9, 44, 46].

Our findings show that migrant youth coming to live with their families had higher odds of engaging in condomless sex in the last year than those migrating as asylum seekers/refugees. This is an unexpected finding since parental and family support and monitoring can be a protective factor against engaging in sexual risk-behaviors [26, 47]. However, we might want to observe this in the context of migrant parents whose financial and social stability in Sweden is not consistent. This might give them less time to deal with family dynamics and provide the needed social support to their children [44].

Belonging to the Islamic religion is a protective factor against condomless sex compared to non-religious or belonging to other faiths. Several studies observed that risk-taking (including sexual risk-taking and extramarital sex) is lower among Muslim people [48–50]. This could be explained as religion-based attitudes, which can reduce the involvement of young people in risky behaviors due to moral teachings, self-control and a sense of value and purpose [51]. Violating these behaviors could jeopardize the family bonds and relationships and generate stigma [52].

Our adjusted estimates show that migrant youth coming from Europe and the MENA region had higher odds of having sex under the influence of drugs in the last year than those coming from Syria. This could be explained by different cultural norms around drug use in youth among countries, which might be continued in Sweden. Although there are no published data on youth use of drugs during sex in Syria, studies in Europe and Africa have found that drug use is frequent among migrants [34, 53, 54]. For example, a multicountry study in Europe found that 70 and 25% of young people have used cannabis and cocaine, respectively, with 26% reporting using it to facilitate sex [53]. In addition, the religious views of Islam as a predominant religion for Syrians are stringent against drug use; therefore, it is unlikely that the prevalence of drugs would be as high as in Europe and Africa [48].

Coming to work/study compared to arriving as an asylum seeker/refugee was a risk factor for engaging in sex under the influence of drugs. This finding aligns with findings from a study conducted in the United States of America [55]. The possibility to live in another place for work/study may increase the sense of liberation and thrill, which could lead to trying new experiences such as having sex under the influence of drugs. Another explanation could be that coming to work/study gives a sense of freedom since there is no parental control, which may prompt engaging in sexual risk-behavior [41]. Lastly, this finding can be further elaborated by the possibility of non-static migration, where migrant youth who work and study might be traveling abroad (or home countries) and engaging in sexual intercourse under the influence of drugs and returning to Sweden [12, 56].

Age was a protective factor for engaging in sex under the influence of drugs. This finding could be explained by the more exploratory nature of youth age and the more precautious older aged who engage less in substance abuse during sex. Another explanation could be that younger people are less able to exercise agency than their older counterparts [57].

Our findings show that the longer migrant youth lived in Sweden the higher odds they had of having sex in exchange for gifts/money in the last year. A possible pathway explaining our findings is acculturation stress theory which hypothesizes that the stressors associated with adaptation to a new country and culture (e.g., discrimination, language barriers, financial instability, cultural clashes, long waiting for the asylum process to be finalized, etc.) make migrant youth highly susceptible to negative coping behaviors such as selling or buying sex [34, 58, 59].

Living with family was another risk factor for engaging in sexual activity in exchange for gifts and money. Migrant youth may engage in such behavior because of a lack of sexual decision-making power and selling sex [60]. An additional explanation could be that some participants belong to sexual and gender minorities, a population of higher vulnerability [30, 61]. Having their family with them could only add to the stress of not having the possibility to be open in their sexual identity which can lead to selling or buying sex [62]. However, this hypothesis cannot be confirmed since we did not collected data on the migrant youth´ sexual orientation.

Lastly, we did not explore the family structure and whether the participants live in both-parent and single-parent households. Research shows that the family structure plays a significant role in modelling personalities and behavior [63].

In this study, arriving to Sweden to work/study compared to arriving as an asylum seeker/refugee was also a risk factor for having sex in exchange for gifts/money. We did not differentiate between selling and buying sex in our survey. Therefore, our findings could have a twofold explanation. One possible pathway is the economic power that can lead to buying sex where buying could be a straightforward path to engage in sexual intercourse. On the other hand, selling sex could be a means of additional income or a way to secure or maintain a job position, as well as means of income due to unemployment [61]. The compensation for sex could be material gifts and alcohol and drugs, suggesting a link for financing substance abuse [64]. Therefore, it is crucial to observe migration status in context.

There was no significant difference in sexual risk-taking behavior between the sexes. In other studies, men had more willingness, tendency and were engaged more frequently in risk behavior than women [51, 65]. Women are perceived to be involved in less sexual risk-taking behaviors, which can be explained by higher adherence to morality, chastity, and family traditions adherence [66].

Our findings could be explained by the impact and adoption of beliefs, norms, and values in the new environment where gender power and differences are less prominent and could influence the possibility of engagement in sexual risk-taking behavior. This could also be explained by a higher degree of monitoring from relatives or response bias due to acculturation in highly restrictive societies where the women’s sexual activity is disparaged [42]. There could also be a difference between countries of origin that place different values on men versus women and thus insert different traditional norms and values (e.g., sexual decision-making power, sexual abstinence before marriage, etc.). However, our finding does not negate that migrant youth and asylum seekers are at risk of experiencing sexual violence during their journey to the host country and at the host country itself, which could add to the sexual risk-taking behavior prevalence, as shown in other studies [67]. These findings are relevant to observe through the context of socioeconomic and structural inequalities migrant youth must endure because they may also affect the possibility of a post-migration acquisition of sexually-transmitted HIV [30]. It is a significant field for further discussion because of an opportunity to set a foundation for healthy sexual and reproductive health choices at an early age with targeted interventions [68].

### Limitations

The language schools and high schools were chosen due to the ease of access to a heterogeneous group of migrants gathered at one place, but asylum-seekers and undocumented migrants are not equally represented there as much as different types of migrants. In the survey, we posed a question on arrival mechanism to Sweden (as an asylum seeker, through work or study-related program, or coming with a family); however, the sensitivity of the question might discourage the respondent from giving an honest answer or skipping the question altogether.

Another limitation of our study is the absence of measurement of other sexual risk-taking behavior variables, such as the number of partners during the last year, early sexual debut, sex with casual partners or concurrent relationships. The reason for this is that we strived to keep the questionnaire concise and straightforward to facilitate data collection. As reflected in our sample, the target population was expected to have a lower educational level; 60% had less than nine years of formal education). Adding more variables (instead of measuring these) could have allowed us to better understand the prevalence of different sexual risk-taking patterns in this population. Thus, it is likely that our sexual risk-taking prevalence among selected behaviors is underestimated. Further studies must evaluate the trade-off between measuring sexual risk-taking using short versus comprehensive scales in populations with low educational levels.

We measured condom use during last year. Although measuring condom use during last intercourse is commonly used, it has the limitation that it only refers to one specific point in time. It has been argued that it might not reflect a person’s long term pattern of condom use, which can be associated with social desirability bias sexual behavior and might overestimate condom use [69]. On the other hand, not measuring other condom related variables such as condom use according to the type of partner (stable, occasional) or frequency of condom use (always, occasionally, rarely, never) did not allow us to evaluate condom use variation according to these variables. Nevertheless, we used the variable “current living arrangements” (Alone, or Married or cohabiting, or With other family, or With friends I knew, or In a refugee home) as a proxy measurement to describe variation in sexual risk-taking behaviors in our sample. This variable provides information about current partner status in Sweden, which might represent more accurately variation in last 12 months sexual risk behaviors.

Another limitation of this study is that we did not measure the sample’s exposure to different forms of violence during their journey to Sweden. This is an important determinant of sexual risk taking among migrant populations. Thus, more studies are needed to map the prevalence of violence exposure in this population and its association with sexual risk taking.

The respondents’ age could not guarantee that they entirely understand their residential status (whether they have a permanent or temporary residence permit) or the reason for coming to Sweden. The questions on sexual risk behavior could be perceived as sensitive and could possibly be underreported. Since we asked questions about last year’s recall, bias could be present. There was a significant underrepresentation of people with low literacy levels in our study. Other types of reach-out structures to gather their opinion could potentially be helpful. Even though there are still contradictions on this issue worth exploring further, both in Sweden and abroad, we reason that these findings are an important alert to policymakers, service providers, and the broader community.

## Conclusion

The heterogeneity in migration pathways to living in Sweden is characterized by changing sexual practices and engaging in sexual intercourse over several years. Sexual risk-taking behavior is an underestimated peril that can have adverse health outcomes in the future, but it requires additional research on the causes. Our study findings highlight the importance of reconsidering the broader system response and ways of delivering the message to migrant youth to safeguard sexual and reproductive health.

Young age is marked with risk behaviors due to maturation processes that are ongoing until adulthood. Being sexually active without reliable information on safe-sex practices could lead to poor choices. Creating programs at an early age of arrival for conveying evidence-based messaging about sexual and reproductive health and rights and long-term consequences of sexual risk-taking behavior in general could have relevant public health implications and prevent detrimental health outcomes.

## Supplementary Information

**Additional file 1.**

**Additional file 2.**

## Data Availability

The data that support the findings of this study are available on request from the project leader and co-author [AME] on reasonable request. The data are not publicly available due to information still being used for publishing two PhD thesis.

## References

[1] Castañeda H, Holmes SM, Madrigal DS, Young M-ED, Beyeler N, Quesada J (2015). Immigration as a Social Determinant of Health. Annu Rev Public Health.

[2] IOM (2020). World Migration Report 2020 [Internet].

[3] UNHCR. Refugee Statistics [Internet]. [cited 2021 May 5]. Available from: https://www.unhcr.org/refugee-statistics/.

[4] Yu B, Chen X, Elliott AL, Wang Y, Li F, Gong J (2019). Social capital, migration stress, depression and sexual risk behaviors among rural-to-urban migrants in China: a moderated mediation modeling analysis. Anxiety Stress Coping.

[5] Chawla N, Sarkar S (2019). Defining “High-risk Sexual Behavior” in the Context of Substance Use. J Psychosex Health.

[6] Strunin L, Hingson R (1992). Alcohol, Drugs, and Adolescent Sexual Behavior. Int J Addict.

[7] Kumari A, Nair R (2012). Predictors of high risk sexual behavior among men in India. J Fam Welf.

[8] Imaledo JA, Peter-Kio OB, Asuquo EO (2012). Pattern of risky sexual behavior and associated factors among undergraduate students of the University of Port Harcourt, Rivers State, Nigeria. Pan Afr Med J.

[9] Pengpid S, Peltzer K (2020). Prevalence and Correlates of Sexual Risk Behavior among School-Going Adolescents in Four Caribbean Countries. Behav Sci.

[10] Igra V, Irwin CE (1996). Theories of Adolescent Risk-Taking Behavior.

[11] Weine S, Bahromov M, Loue S, Owens L (2013). HIV sexual risk behaviors and multilevel determinants among male labor migrants from Tajikistan. J Immigr Minor Health.

[12] Dias S, Gama A, Loos J, Roxo L, Simões D, Nöstlinger C (2020). The role of mobility in sexual risk behaviour and HIV acquisition among sub-Saharan African migrants residing in two European cities. Caylà JA, editor. PLoS One.

[13] McGrath N, Hosegood V, Newell ML, Eaton JW (2015). Migration, sexual behaviour, and HIV risk: A general population cohort in rural South Africa. Lancet HIV.

[14] Olawore O, Tobian AAR, Kagaayi J, Bazaale JM, Nantume B, Kigozi G (2018). Migration and risk of HIV acquisition in Rakai, Uganda: a population-based cohort study. Lancet HIV.

[15] Finger S (2016). Sex-work and mobility as a coping strategy for marginalized Hungarian Roma women. Acme..

[16] Hjern A (2012). Migration and public health: Health in Sweden: The National Public Health Report 2012. Chapter 13. Scand J Public Health.

[17] Statistikmyndigheten SCB (2021). Utrikes födda i Sverige [Internet].

[18] Migrationsverket (2016). Applications for asylum received [Internet].

[19] Migrationsverket (2021). Health care for asylum seekers [Internet].

[20] Svensson P, Carlzén K, Agardh A (2017). Exposure to culturally sensitive sexual health information and impact on health literacy: a qualitative study among newly arrived refugee women in Sweden. Culture Health Sexuality.

[21] Skolverket. Sex and cohabitation education - an introduction. Gender equality, sexuality and relationships in the curricula [Internet]. 2019 [cited 2021 Oct 12]. Available from: https://www.skolverket.se/skolutveckling/inspiration-och-stod-i-arbetet/stod-i-arbetet/sex-och-samlevnad.

[22] United Nations. About the Sustainable Development Goals - United Nations Sustainable Development [Internet]: Sustainable Development Goals; 2015. p. 1. Available from: https://www.un.org/sustainabledevelopment/sustainable-development-goals/

[23] Baroudi M, Kalengayi FN, Goicolea I, Jonzon R, Sebastian MS, Hurtig A-K. Access of Migrant Youths in Sweden to Sexual and Reproductive Healthcare: A Cross-sectional Survey. Int J Health Policy Manage. 2020; [cited 2021 May 10]; Available from: https://pubmed.ncbi.nlm.nih.gov/32729283/. 10.34172/ijhpm.2020.123 PMC9278465 32729283

[24] Helgesson M, Johansson B, Nordquist T, Vingård E, Svartengren M (2019). Healthy migrant effect in the Swedish context: a register-based, longitudinal cohort study. BMJ Open.

[25] Folkhälsomyndigheten (2019). Hälsa hos personer som är utrikes födda – skillnader i hälsa utifrån födelseland.

[26] Asamoah BO, Agardh A (2018). Individual- and Family-Level Determinants of Risky Sexual Behavior Among Swedish- and Foreign-Born Young Adults 18–30 Years of Age, Residing in Skåne, Sweden. Arch Sex Behav.

[27] IOM (2019). Glossary on migration [Internet].

[28] Karolinska Institutet. Research Projects | Global and Sexual Health [Internet]. [cited 2021 May 5]. Available from: https://ki.se/en/gph/research-projects-global-and-sexual-health.

[29] Brännström J, Sönnerborg A, Svedhem V, Neogi U, Marrone G (2017). A high rate of HIV-1 acquisition post immigration among migrants in Sweden determined by a CD4 T-cell decline trajectory model. HIV Med.

[30] Alvarez-Del Arco D, Fakoya I, Thomadakis C, Pantazis N, Touloumi G, Gennotte A-F (2017). High levels of postmigration HIV acquisition within nine European countries. AIDS (London, England).

[31] Migrationsverket (2021). Asyl [Internet].

[32] Rubin DB. Basic Ideas of Multiple Imputation for Nonresponse [Internet]. 12, . 1986. p. 37–47. Survey Methodology. Available from: https://www150.statcan.gc.ca/n1/en/pub/12-001-x/1986001/article/14439-eng.pdf?st=6eB6lSXd

[33] Folkhälsomyndigheten. Migration, sexuell hälsa och hiv/STI prevention - en sammanfattande rapport [Internet]. Stockholm; 2020. Available from: https://www.folkhalsomyndigheten.se/publicerat-material/publikationsarkiv/m/migration-sexuell-halsa-och-hivsti-prevention/?pub=68937

[34] Eubanks A, Parriault MC, van Melle A, Basurko C, Adriouch L, Cropet C (2018). Factors associated with sexual risk taking behavior by precarious urban migrants in French Guiana. BMC Int Health Hum Rights.

[35] Folkhälsomyndigheten (2017). Sexuality and health among young people in Sweden [Internet].

[36] Helsing B, Frisén A, Hwang CP (2021). Sexual risk-taking among young Swedish men testing for STI. Eur J Contracept Reprod Health Care.

[37] Dias S, Marques A, Gama A, Martins M (2014). HIV Risky Sexual Behaviors and HIV Infection Among Immigrants: A Cross-Sectional Study in Lisbon, Portugal. Int J Environ Res Public Health.

[38] Gras MJ, van Benthem BHB, Coutinho RA, van den Hoek A (2001). Determinants of High-Risk Sexual Behavior Among Immigrant Groups in Amsterdam: Implications for Interventions. J Acquir Immune Defic Syndr.

[39] Burns FM, Evans AR, Mercer CH, Parutis V, Gerry CJ, Mole RCM (2011). Sexual and HIV risk behaviour in Central and Eastern European migrants in London. Sex Transm Infect.

[40] Larsson A. Assessing Sexual Risk Among Youth In Sweden: Does Immigrant Status Matter?, 2012. [Internet]. Available from: https://www.researchgate.net/publication/313376286_Assessing_Sexual_Risk_Among_Youth_In_Sweden_Does_Immigrant_Status_Matter.

[41] Muindi K, Mudege N, Beguy D, Mberu BU (2014). Migration and sexual behaviour among youth in Nairobi’s slum areas. Afr Popul Stud.

[42] Meston CM, Ahrold T (2010). Ethnic, Gender, and Acculturation Influences on Sexual Behaviors. Arch Sex Behav.

[43] Fakoya I, Álvarez-del Arco D, Woode-Owusu M, Monge S, Rivero-Montesdeoca Y, Delpech V (2015). A systematic review of post-migration acquisition of HIV among migrants from countries with generalised HIV epidemics living in Europe: mplications for effectively managing HIV prevention programmes and policy. BMC Public Health.

[44] Osman F, Mohamed A, Warner G, Sarkadi A (2020). Longing for a sense of belonging—Somali immigrant adolescents’ experiences of their acculturation efforts in Sweden. Int J Qual Stud Health Well Being.

[45] Johansson S, Danielsson VJ (2016). En invandrares nya identitet i Sverige [Internet].

[46] Herlitz C (2009). Sexual risk-taking in the general population of Sweden (1989-2007). Sex Health.

[47] Yi S, Te V, Pengpid S, Peltzer K (2018). Social and behavioural factors associated with risky sexual behaviours among university students in nine ASEAN countries: a multi-country cross-sectional study. SAHARA-J J Soc Asp HIV AIDS.

[48] Curtis P, Thompson J, Fairbrother H (2018). Migrant children within Europe: a systematic review of children’s perspectives on their health experiences. Public Health.

[49] Smith C, Faris R (2002). Religion and American Adolescent Delinquency, Risk Behaviors and Constructive Social Activities. A Research Report of the National Study of Youth and Religion. Tradition A J Orthodox Jew Thought.

[50] Adamczyk A, Hayes BE (2012). Religion and Sexual Behaviors. Am Sociological Rev.

[51] Ameri Z, Mirzakhani F, Nabipour AR, Khanjani N, Sullman MJM (2017). The Relationship Between Religion and Risky Behaviors Among Iranian University Students. J Relig Health.

[52] Abu-Ras W, Ahmed S, Arfken CL (2010). Alcohol use among U.S. Muslim college students: risk and protective factors. J Ethn Subst Abuse.

[53] Bellis MA, Hughes K, Calafat A, Juan M, Ramon A, Rodriguez JA (2008). Sexual uses of alcohol and drugs and the associated health risks: A cross sectional study of young people in nine European cities. BMC Public Health.

[54] Olawole-Isaac A, Ogundipe O, Amoo EO, Adeloye DO (2018). Substance use among adolescents in sub-Saharan Africa: a systematic review and meta-analysis. South Afr J Child Health.

[55] Paat Y-F, Torres LR, Morales DX, Srinivasan SM, Sanchez S (2020). Sensation seeking and impulsivity as predictors of high-risk sexual behaviours among international travellers. Curr Issues Tourism.

[56] Persson KI, Berglund T, Bergström J, Tikkanen R, Thorson A, Forsberg B (2018). Place and practice: Sexual risk behaviour while travelling abroad among Swedish men who have sex with men. Travel Med Infect Dis.

[57] Zimmerman LA, Li M, Moreau C, Wilopo S, Blum R (2019). Measuring agency as a dimension of empowerment among young adolescents globally; findings from the Global Early Adolescent Study. SSM Popul Health.

[58] Wrede O, Löve J, Jonasson JM, Panneh M, Priebe G (2021). Promoting mental health in migrants: a GHQ12-evaluation of a community health program in Sweden. BMC Public Health.

[59] Okenwa-Emegwa L, Saboonchi F, Mittendorfer-Rutz E, Helgesson M, Tinghög P (2019). Prevalence and predictors of low future expectations among Syrian refugees resettled in Sweden. Heliyon.

[60] Du H, Li X (2015). Acculturation and HIV-related sexual behaviours among international migrants: a systematic review and meta-analysis. Health Psychol Rev.

[61] Alessi EJ, Kahn S, Giwa S, Cheung S (2020). “Those tablets, they are finding an empty stomach”: a qualitative investigation of HIV risk among sexual and gender minority migrants in Cape Town, South Africa. Ethn Health.

[62] Sandfort T, Anyamele C, Dolezal C (2017). Correlates of Sexual Risk among Recent Gay and Bisexual Immigrants from Western and Eastern Africa to the USA. J Urban Health.

[63] Mmari K, Cooper D, Moreau C, Koenig L, Martinez M, Mafuta E (2021). The Social Context of Early Adolescents in the Global Early Adolescent Study. J Adolesc Health.

[64] Krisch M, Averdijk M, Valdebenito S, Eisner M (2019). Sex Trade Among Youth: A Global Review of the Prevalence, Contexts and Correlates of Transactional Sex Among the General Population of Youth. Adolesc Res Rev.

[65] Bradley G, Wildman K (2002). Psychosocial Predictors of Emerging Adults’ Risk and Reckless Behaviors. J Youth Adolesc.

[66] Munro-Kramer ML, Fava NM, Saftner MA, Darling-Fisher CS, Tate NH, Stoddard SA (2016). What are we missing? Risk behaviors among Arab-American adolescents and emerging adults. J Am Assoc Nurse Pract.

[67] United Nations Department of Economic and Social Affairs. Youth and Migration [Internet], vol. 2013: World youth report; 2013. Available from: http://www.un.org/esa/socdev/documents/youth/fact-sheets/youth-migration.pdf

[68] Woog V, Kagesten A. The Sexual and Reproductive Health Needs of Very Young Adolescents Aged 10–14 in Developing Countries: What Does the Evidence Show? Guttmacher Inst. 2017;(May):1–56 Available from: https://www.guttmacher.org/report/srh-needs-very-young-adolescents-in-developing-countries.

[69] Fonner VA, Kennedy CE, O’Reilly KR, Sweat MD. Systematic assessment of condom use measurement in evaluation of HIV prevention interventions: need for standardization of measures. AIDS Behav. 2014;18(12):2374–86 Available from: http://link.springer.com/10.1007/s10461-013-0655-1. 10.1007/s10461-013-0655-1 PMC4013255 24197972

