# Supplementary material for: Sexual risk-taking behaviors among young migrant population in Sweden
Source: BMC Public Health. 2022 Mar 30;22:625. doi: 10.1186/s12889-022-12996-2 (PMC8969344; doi:10.1186/s12889-022-12996-2)
Supplement: Supplementary file 1 — Additional file 1. [file 12889_2022_12996_MOESM1_ESM.docx]

**Additional file 1**

**Descriptive statistics of sociodemographic characteristics by main outcome variables among 1,563 participants (15-25 years old)**

| **Characteristics** | **All participants** | **Having sex**  **without a condom** | | **Influence of drugs during sex** | | **Sex in exchange for gifts/money** | |
| --- | --- | --- | --- | --- | --- | --- | --- |
|  |  | **No** | **Yes** | **No** | **Yes** | **No** | **Yes** |
|  | n (%) | n (%) | n (%) | n (%) | n (%) | n (%) | n (%) |
| **Sex** |  |  |  | * | * |  |  |
| Male | 856 (55.8) | 247 (62.7) | 134 (63.2) | 357 (55.5) | 37 (74.0) | 366 (56.6) | 32 (64.0) |
| Female | 677 (44.2) | 147 (37.3) | 78 (36.8) | 286 (44.5) | 13 (26.0) | 281 (43.4) | 18 (36.0) |
| Total | *1533 (100)* | *394 (100)* | *212 (100)* | *643 (100)* | *50 (100)* | *647 (100)* | *50 (100)* |
| **Age (Mean (SD))** | 19.65 (2.7) | 19.4 (2.64) | 20.1 (2.69) | 19.7 (2.73) | 18.8 (2.05) | 19.7 (2.73) | 19.4 (2.23) |
| **Education (years)** |  |  |  |  |  |  | |
| Less than 3 | 341 (23.2) | 84 (21.6) | 48 (23.2) | 141 (22.0) | 10 (20.4) | 139 (21.7) | 10 (20.8) |
| 4-6 | 336 (22.9) | 92 (23.6) | 44 (21.3) | 139 (21.7) | 12 (24.5) | 143 (22.3) | 12 (25.0) |
| 7-9 | 181 (12.3) | 65 (16.7) | 24 (11.6) | 90 (14.1) | 11 (22.4) | 90 (14.0) | 6 (12.5) |
| 10 or longer | 609 (41.5) | 148 (38.0) | 91 (43.9) | 270 (42.2) | 16 (32.6) | 269 (41.9) | 20 (41.9) |
| Total | *1467 (100)* | *389 (100)* | *207 (100)* | *640 (100)* | *49 (100)* | *641 (100)* | *48 (100)* |
| **Living in Sweden (years) (Mean (SD))** | 2.50 (1.52) | 2.54 (1.50) | 2.70 (1.53) | 2.55 (1.51) | 2.9 (1.43) | 2.58 (1.52) | 2.76 (1.36) |
| **Religion** |  | *** | *** |  |  |  | |
| Not religious, other | 231 (18.5) | 69 (17.6) | 49 (23.8) | 105 (16.5) | 14 (28.6) | 112 (17.5) | 11 (23.4) |
| Christianity | 254 (20.4) | 78 (19.8) | 52 (25.2) | 126 (19.8) | 11 (22.4) | 127 (19.8) | 13 (27.6) |
| Islam | 762 (61.1) | 246 (62.6) | 105 (50.9) | 405 (63.6) | 24 (48.9) | 402 (62.7) | 23 (48.9) |
| Total | *1247 (100)* | *393 (100)* | *206 (100)* | *636 (100)* | *49 (100)* | *641 (100)* | *47 (100)* |
| **Country/born and raised** |  | *** | *** | *** | *** |  |  |
| Australia, Asia, Other | 92 (6.25) | 21 (5.5) | 8 (3.9) | 29 (4.61) | 3 (6.2) | 32 (5.1) | 3 (6.4) |
| Americas | 183 (12.4) | 35 (9.1) | 36 (17.8) | 68 (10.8) | 6 (12.5) | 67 (10.6) | 5 (10.6) |
| Europe | 115 (7.81) | 31 (8.1) | 31 (8.1) | 52 (8.3) | 9 (18.7) | 54 (8.5) | 8 (17.0) |
| Africa | 326 (22.1) | 79 (20.6) | 28 (13.9) | 131 (20.8) | 5 (10.4) | 124 (19.6) | 10 (21.3) |
| MENA countries** | 190 (12.9) | 46 (12.0) | 31 (15.3) | 77 (12.2) | 9 (18.7) | 80 (12.7) | 5 (10.6) |
| Syria | 325 (22.0) | 97 (25.3) | 34 (16.8) | 165 (26.2) | 4 (8.3) | 161 (25.5) | 6 (12.8) |
| Afghanistan | 242 (16.4) | 74 (19.3) | 34 (16.8) | 107 (17.0) | 12 (25.0) | 114 (18.0) | 10 (21.3) |
| Total | *1473 (100)* | *383 (100)* | *202 (100)* | *629 (100)* | *48 (100)* | *632 (100)* | *47 (100)* |
| **Having a residence permit in Sweden** |  |  |  | *** | *** |  |  |
| No | 106 (9.5) | 28 (9.9) | 14 (9.7) | 41 (8.7) | 8 (25.0) | 39 (8.3) | 4 (11.8) |
| Yes | 967 (86.8) | 239 (84.7) | 125 (86.2) | 411 (87.3) | 22 (68.7) | 411 (87.4) | 29 (85.3) |
| I am an EU/EEA/Swedish citizen | 41 (3.7) | 15 (5.3) | 6 (4.1) | 19 (4.0) | 6.2) | 20 (4.3) | 1 (2.9) |
| Total | *1114 (100)* | *282 (100)* | *145 (100)* | *471 (100)* | *32 (100)* | *470 (100)* | *34 (100)* |
| **The main reason to come to Sweden** |  |  |  | *** | *** | *** | *** |
| As an asylum seeker/refugee | 821 (61.2) | 240 (67.0) | 107 (58.1) | 372 (64.8) | 24 (54.5) | 379 (65.7) | 22 (55.0) |
| To work/study | 157 (11.7) | 43 (12.0) | 30 (16.3) | 58 (10.1) | 15 (34.0) | 64 (11.1) | 11 (27.5) |
| To live with family | 260 (19.4) | 58 (16.2) | 35 (19.0) | 103 (17.9) | 3 (6.8) | 101 (17.5) | 6 (15.0) |
| Other | 103 (7.7) | 17 (4.7) | 12 (6.5) | 41 (7.2) | 2 (4.5) | 33 (5.7) | 1 (2.5) |
| Total | *1341 (100)* | *358 (100)* | *184 (100)* | *574 (100)* | *618 (100)* | *577 (100)* | *40 (100)* |
| **Current living arrangements** |  | *** | *** | *** | *** |  |  |
| Alone | 275 (20.8) | 87 (21.9) | 52 (25.0) | 126 (19.6) | 17 (34.7) | 129 (20.0) | 13 (26.5) |
| Married or cohabiting | 169 (12.8) | 15 (10.1) | 37 (17.9) | 93 (14.4) | 1 (2.0) | 93 (14.4) | 3 (6.1) |
| With other family | 726 (54.9) | 214 (53.9) | 96 (46.1) | 338 (52.5) | 26 (53.1) | 339 (52.6) | 30 (61.2) |
| With friends I knew from earlier | 117 (8.8) | 47 (11.8) | 15 (7.2) | 66 (10.2) | 4 (8.2) | 66 (10.2) | 2 (4.1) |
| In a refugee home | 36 (2.7) | 9 (2.3) | 8 (3.8) | 21 (3.2) | 1 (2.0) | 18 (2.8) | 1 (2.0) |
| Total | *1323 (100)* | *397 (100)* | *208 (100)* | *644 (100)* | *49 (100)* | *645 (100)* | *49 (100)* |

*Comparison between those reporting any sexual risk-taking or not, *p* < 0.05, chi2 test

**MENA countries (Algeria, Bahrain, Egypt, Iran, Iraq, Israel, Jordan, Kuwait, Lebanon, Libya, Morocco, Oman, Qatar, Saudi Arabia, Palestine, Syria (excluded), Tunisia, United Arab Emirates, Yemen)
