# Supplementary material for: Sexual risk-taking behaviors among young migrant population in Sweden
Source: BMC Public Health. 2022 Mar 30;22:625. doi: 10.1186/s12889-022-12996-2 (PMC8969344; doi:10.1186/s12889-022-12996-2)
Supplement: Supplementary file 2 — Additional file 2. [file 12889_2022_12996_MOESM2_ESM.docx]

**Additional file 2**

**Sensitivity analysis**

**Table 1.** **Association between condomless sex in the last year, demographic characteristics and migration-related variables, adjusted odds ratios (AOR) and 95% confidence intervals (CI) shown**

| **Characteristics** | **Model 1**  **(n=471)** | **Model 2**  **(n=427)** | **Model 3**  **(n=327)** | **Model 4**  **(n=462)** | **Model 5**  **(n=311)** | **Model 6**  **(n=1394)** |
| --- | --- | --- | --- | --- | --- | --- |
|  | **AOR (95%CI)** | **AOR 95% CI** | **AOR 95% CI** | **AOR 95%CI** | **AOR 95%CI** | **AOR 95%CI** |
| **Sex (ref: Males)** | 1.00 (reference) | | | | | |
| Females | 0.87 (0.55-1.38) | 0.73 (0.43-1.23) | 0.91 (0.51-1.64) | 0.89 (0.54-1.46) | 0.93 (0.47-1.85) | 0.85 (0.54-1.36) |
| ***Age (years)** | 1.00 (reference) | | | | | |
| *Mean and SD | 1.08 (0.99-1.18) | 1.10 (1.00-1.21) * | 1.09 (0.98-1.22) | 1.09 (0.99-1.20) | 1.09 (0.96-1.22) | 1.12 (1.03-1.22)** |
| **Education (years)**  **(ref: less than 3 years)** | 1.00 (reference) | | | | | |
| 4-6 | 0.75 (0.41-1.38) | 0.73 (0.39-1.35) | 0.87 (0.44-1.73) | 0.78 (0.41-1.45) | 0.85 (0.41-1.76) | 0.73 (0.41-1.29) |
| 7-9 | 0.82 (0.41-1.66) | 0.76 (0.34-1.67) | 0.91 (0.34-2.46) | 0.84 (0.41-1.74) | 1.16 (0.39-3.47) | 0.66 (0.36-1.24) |
| 10 or longer | 0.77 (0.40-1.48) | 0.72 (0.36-1.43) | 0.92 (0.43-1.99) | 0.87 (0.44-1.71) | 1.07 (0.47-2.43) | 0.76 (0.39-1.45) |
| ***Religion (ref: other religion, atheist)** | 1.00 (reference) | | | | | |
| Christianity | 1.08 (0.56-2.07) | 1.11 (0.54-2.29) | 1.23 (0.52-2.93) | 1.09 (0.56-2.14) | 1.01 (0.41-2.50) | 0.87 (0.43-1.76) |
| *Islam | 0.61 (0.35-1.06) | 0.66 (0.35-1.22) | 0.63 (0.30-1.32) | 0.56 (0.31-0.99)* | 0.45 (0.20-0.99)* | 0.56 (0.32-0.99)* |
| ***Living in Sweden (years)** Mean and SD |  |  |  |  |  |  |
|  | 1.21 (1.01-1.43) * | 1.26 (1.04-1.52) * | 1.26 (1.02-1.54)* | 1.30 (1.08-1.55)** | 1.49(1.17-1.89)** | 1.24 (1.05-1.47)* |
| ***Country/Born and raised (ref: Syria)** | 1.00 (reference) | | | | | |
| Other, Australia, Asia | 1.08 (0.39-3.09) | 0.38 (0.09-1.58) | 0.66 (0.16-2.73) | 1.11 (0.38-3.22) | 0.30 (0.05-1.63) | 0.72 (0.21-2.42) |
| *Americas | 2.67 (1.29-5.55)** | 1.97 (0.90-4.30) | 1.52 (0.67-3.42) | 2.74 (1.30-5.80)** | 1.29 (0.54-3.09) | 2.21 (0.98-5.01) |
| Europe | 1.97 (1.86-4.50) | 1.11 (0.41-3.03) | 1.23 (0.33-4.63) | 2.31 (0.96-5.53) | 0.78 (0.15-5.90) | 1.98 (0.93-4.15) |
| Africa | 0.69 (0.34-1.41) | 0.68 (0.32-1.42) | 0.59 (0.25-1.38) | 0.65 (0.31-1.39) | 0.50 (0.19-1.24) | 0.75 (0.40-1.43) |
| MENA countries | 1.30 (0.62-2.72) | 1.23 (0.56-2.72) | 0.71 (0.28-1.78) | 1.26 (0.58-2.76) | 0.52 (0.18-1.49) | 1.15 (0.49-2.73) |
| *Afghanistan | 0.75 (0.37-1.53) | 0.67 (0.32-1.41) | 0.44 (0.18-1.03) | 0.67 (0.31-1.46) | 0.32 (0.12-0.84)* | 0.76 (0.37-1.57) |
| ***Main reason to come to Sweden**  **(ref: As an asylum seeker/refugee)** | 1.00 (reference) | | | | | |
| *To work/study |  | 2.43 (1.13-5.22)* |  |  | 2.98 (1.02-8.74)* | 1.77 (0.83-3.82) |
| To live with family |  | 1.62 (0.79-3.37) |  |  | 1.33 (0.58-3.01) | 1.18 (0.53-2.60) |
| Other |  | 1.65 (0.58-4.76) |  |  | 2.84 (0.86-9.41) | 2.04 (0.84-4.97) |
| **Having a residence permit (ref: EU/EEA/Swedish citizen)** | 1.00 (reference) | | | | | |
| No |  |  | 2.94 (0.51-17.07) |  | 2.76 (0.40-19.11) | 2.25 (0.47-10.69) |
| Yes |  |  | 2.78 (0.50-15.45) |  | 3.16 (0.47-21.37) | 1.92 (0.54-6.84) |
| **Current living arrangements**  **(ref: with other family)** | 1.00 (reference) | | | | | |
| Alone |  |  |  | 1.14 (0.65-1.97) | 1.41 (0.67-2.96) | 1.27 (0.81-1.99) |
| With friends I knew from earlier |  |  |  | 0.56 (0.27-1.18) | 0.88 (0.36-2.12) | 0.51 (0.22-1.17) |
| In a refugee home |  |  |  | 2.67 (0.83-8.64) | 1.33 (0.25-7.03) | 1.85 (0.58-5.88) |

Legend: * p<0.05; ** p<0.01

*MENA countries (Algeria, Bahrain, Egypt, Iran, Iraq, Israel, Jordan, Kuwait, Lebanon, Libya, Morocco, Oman, Qatar, Saudi Arabia, Palestine, Syria (excluded), Tunisia, United Arab Emirates, Yemen)

Crude OR for considered explanatory factors.

Model 1, Adj. OR for sociodemographic factors: sex, age, country born or raised, religion, number of years living in Sweden, education, Model 2, Adj., OR factors included in Model 1 + Main reason to come to Sweden; Model 3 included Model 1+ Having a residence permit in Sweden; Model 4 included Model 1+Current living arrangements. Model 5 included all variables. Model 6 is a multiple imputation model.

**Table 2. Association between having sex under the influence of drugs in the last year, demographic characteristics and migration-related variables, adjusted odds ratios (AOR) and 95% confidence intervals (CI) shown**

| **Characteristics** | **Model 1**  **(n=537)** | **Model 2**  **(n=479)** | **Model 3**  **(n=375)** | **Model 4**  **(n=525)** | **Model 5**  **(n=351)** | **Model 6**  **(n=1394)** |
| --- | --- | --- | --- | --- | --- | --- |
|  | **AOR (95%CI)** | **AOR 95% CI** | **AOR 95% CI** | **AOR 95%CI** | **AOR 95%CI** | **AOR 95%CI** |
| **Sex (ref: Males)** | 1.00 (reference) | | | | | |
| Females | 0.52 (0.23-1.14) | 0.40 (0.15-1.07) | 0.34 (0.01-1.10) | 0.57 (0.25-1.30) | 0.31 (0.07-1.27) | 0.93 (0.29-2.89) |
| **Age (years)**  Mean and SD | 1.00 (reference) | | | | | |
|  | 0.90 (0.77-1.06) | 0.87 (0.72-1.03) | 0.90 (0.72-1.12) | 0.92 (0.78-1.09) | 0.86 (0.66-1.12) | 0.88 (0.76-1.01) |
| **Education (years)**  **(ref: less than 3 years)** | 1.00 (reference) | | | | | |
| 4-6 | 1.46 (0.53-3.99) | 1.68 (0.58-4.87) | 1.80 (0.56-5.73) | 1.42 (0.48-4.14) | 1.67 (0.47-5.91) | 1.41 (0.42-4.66) |
| 7-9 | 2.05 (0.69-6.11) | 1.85 (0.52-6.51) | 2.02 (0.46-8.92) | 2.27 (0.71-7.19) | 2.45 (0.47-12.56) | 1.23 (0.39-4.19) |
| 10 or longer | 1.58 (0.50-4.94) | 2.03 (0.59-6.90) | 2.10 (0.55-8.03) | 1.75 (0.53-5.76) | 2.38 (0.54-10.58) | 1.25 (0.37-4.20) |
| **Religion (ref: other religion, atheist)** | 1.00 (reference) | | | | | |
| Christianity | 1.28 (0.47-3.44) | 0.91 (0.30-2.76) | 0.81 (0.20-3.29) | 1.40 (0.50-3.89) | 0.72 (0.16-3.19) | 1.23 (0.51-2.91) |
| Islam | 0.65 (0.29-1.49) | 0.67 (0.27-1.67) | 0.75 (0.26-2.11) | 0.58 (0.25-1.35) | 0.54 (0.17-1.68) | 0.73 (0.29-1.92) |
| **Living in Sweden (years)**  Mean and SD |  |  |  |  |  |  |
|  | 1.25 (0.93-1.68) | 1.34 (0.96-1.88) | 0.99 (0.69-1.43) | 1.27 (0.93-1.72) | 1.11 (0.72-1.69) | 1.33 (0.89-1.97) |
| ***Country/Born and raised (ref: Syria)** | 1.00 (reference) | | | | | |
| Other, Australia, Asia | 4.22 (0.75-23.83) | 0.98 (0.08-11.75) | 1.90 (0.21-16.90) | 4.23 (0.74-24.07) | 0.39 (0.02-7.08) | 1.96 (0.15-25.84) |
| Americas | 3.72 (0.82-16.89) | 2.25 (0.44-11.47) | 1.49 (0.25-8.83) | 3.39 (0.73-15.75) | 1.15 (0.17-7.75) | 1.75 (0.34-8.94) |
| *Europe | 9.44 (2.15-41.50)** | 7.67 (1.46-40.05)* | 2.42 (0.31-18.88) | 7.68 (1.68-34.99)** | 0.94 (0.07-12.18) | 3.49 (0.57-21.30) |
| Africa | 1.20 (0.24-5.95) | 1.53 (0.30-7.70) | 1.34 (0.23-7.68) | 1.09 (0.21-5.62) | 1.32 (0.21-8.22) | 0.81 (0.17-3.93) |
| *MENA | 5.30 (1.31-21.48)* | 5.17 (1.16-23.09)* | 4.53 (0.96-21.39) | 5.69 (1.37-23.53)* | 4.31 (0.78-23.72) | 2.79 (0.56-13.86) |
| Afghanistan | 3.42 (0.84-13.92) | 3.67 (0.85-15.87) | 2.73 (0.61-12.33) | 3.50 (0.81-15.21) | 2.22 (0.43-11.55) | 2.47 (0.47-12.85) |
| ***Main reason to come to Sweden**  **(ref: As an asylum seeker/refugee)** | 1.00 (reference) | | | | | |
| *To work/study |  | 4.75 (1.59-14.08)** |  |  | 3.28 (0.71-15.22) | 2.96 (1.23-7.20)* |
| To live with family |  | 1.96 (0.45-8.42) |  |  | 2.29 (0.46-11.48) | 0.75 (0.18-3.11) |
| Other |  | 0.62 (0.07-5.25) |  |  | 0.83 (0.09-7.68) | 1.22 (0.26-5.73) |
| **Having a residence permit**  **(ref:EU/EEA/Swedish citizen)** | 1.00 (reference) | | | | | |
| No |  |  | 2.56 (0.19-33.98) |  | 1.49 (0.08-27.43) | 1.53 (0.23-10.21) |
| Yes |  |  | 0.77 (0.06-9.94) |  | 0.57 (0.03-10.12) | 0.81 (0.09-7.15) |
| **Current living arrangements**  **(ref: with other family)** | 1.00 (reference) | | | | | |
| Alone |  |  |  | 1.20 (0.50-2.89) | 1.85 (0.52-6.56) | 1.60 (0.62-4.12) |
| With friends I knew from earlier |  |  |  | 0.61 (0.18-2.03) | 1.23 (0.28-5.24) | 0.63 (0.15-2.62) |
| In a refugee home |  |  |  | 0.53 (0.05-4.82) | 1.25 (0.09-17.38) | 0.31 (0.03-2.78) |

Legend: * p<0.05; ** p<0.01

*MENA countries (Algeria, Bahrain, Egypt, Iran, Iraq, Israel, Jordan, Kuwait, Lebanon, Libya, Morocco, Oman, Qatar, Saudi Arabia, Palestine, Syria (excluded), Tunisia, United Arab Emirates, Yemen)

Crude OR for considered explanatory factors. Model 1, Adj. OR for sociodemographic factors: sex, age, country born or raised, religion, number of years living in Sweden, education. Model 2, Adj., OR factors included in Model 1 + Main reason to come to Sweden; Model 3 included Model 1+ Having a residence permit in Sweden; Model 4 included Model 1+Current living arrangements. Model 5 included all variables. Model 6 is a multiple imputation model.

**Table 3. Association between having exchanged sex for gifts/money in the last year, demographic characteristics and migration-related variables, adjusted odds ratios (AOR) and 95% confidence intervals (CI) shown**

| **Characteristics** | **Model 1**  **(n=537)** | **Model 2**  **(n=456)** | **Model 3**  **(n=362)** | **Model 4**  **(n=509)** | **Model 5**  **(n=306)** | **Multiple imputations**  **(n=1394)** |
| --- | --- | --- | --- | --- | --- | --- |
|  | **AOR (95%CI)** | **AOR 95% CI** | **AOR 95% CI** | **AOR 95%CI** | **AOR 95%CI** | **AOR 95%CI** |
| **Sex (ref: Males)** | 1.00 (reference) | | | | | |
| Females | 0.82 (0.38-1.76) | 0.70 (0.28-1.69) | 0.71 (0.26-1.92) | 0.88 (0.39-1.98) | 0.68 (0.20-2.27) | 1.03 (0.45-2.37) |
| **Age (years)** | 1.00 (reference) | | | | | |
| Mean and SD | 1.00 (0.86-1.15) | 1.01 (0.86-1.18) | 1.10 (0.91-1.33) | 1.00 (0.85-1.16) | 1.11 (0.90-1.37) | 1.01 (0.91-1.12) |
| **Education (years)**  **(ref: less than 3 years)** | 1.00 (reference) | | | | | |
| 4-6 | 1.36 (0.48-3.85) | 1.48 (0.49-4.47) | 1.92 (0.59-6.15) | 1.79 (0.56-5.69) | 2.08 (0.58-7.62) | 1.15 (0.37-3.55) |
| 7-9 | 0.96 (0.26-3.46) | 0.52 (0.09-2.99) | 0.48 (0.05-4.72) | 1.34 (0.33-5.47) | 0.82 (0.07-9.94) | 0.68 (0.15-3.03) |
| 10 or longer | 1.17 (0.36-3.74) | 1.46 (0.42-5.10) | 1.78 (0.4-7.09) | 1.82 (0.51-6.51) | 2.92 (0.63-13.49) | 0.88 (0.32-2.43) |
| **Religion (ref: other religion, atheist)** | 1.00 (reference) | | | | | |
| Christianity | 1.73 (0.59-5.10) | 1.19 (0.36-3.93) | 0.67 (0.14-3.07) | 2.19 (0.68-7.04) | 0.41 (0.08-2.15) | 1.40 (0.43-4.56) |
| Islam | 0.75 (0.29-1.98) | 0.73 (0.25-2.14) | 0.57 (0.17-1.89) | 0.78 (0.28-2.18) | 0.28 (0.07-1.08) | 0.86 (0.41-1.78) |
| **Living in Sweden (years)** Mean and SD |  |  |  |  |  |  |
|  | 1.18 (0.88-1.57) | 1.27 (0.91-1.76) | 1.20 (0.85-1.69) | 1.30 (0.96-1.76) | 1.54 (0.98-2.39) | 1.17 (0.86-1.59) |
| **Country/Born and raised (ref: Syria)** | 1.00 (reference) | | | | | |
| Other, Australia, Asia | 2.85 (0.57-14.13) | 0.64 (0.06-7.08) | 1.78 (0.24-13.18) | 3.15 (0.62-16.02) | 0.22 (0.01-3.55) | 1.93 (0.23-16.22) |
| Americas | 1.56 (0.38-6.39) | 0.91 (0.19-4.31) | 1.16 (0.23-5.64) | 1.57 (0.37-6.59) | 0.67 (0.12-3.84) | 1.23 (0.33-4.54) |
| Europe | 3.32 (0.83-13.27) | 1.72 (0.32-9.02) | 1.58 (0.12-20.32) | 3.20 (0.73-13.89) | (empty) | 3.27 (0.74-14.44) |
| Africa | 1.12 (0.31-4.06) | 1.50 (0.39-5.71) | 1.77 (0.43-7.20) | 1.13 (0.29-4.36) | 1.80 (0.38-8.35) | 1.44 (0.36-5.78) |
| MENA | 1.48 (0.36-6.01) | 1.32 (0.27-6.45) | 0.77 (0.13-4.59) | 1.86 (0.44-7.88) | 0.64 (0.09-4.69) | 1.28 (0.38-4.32) |
| Afghanistan | 1.37 (0.37-5.02) | 1.54 (0.38-6.18) | 1.31 (0.30-5.68) | 1.76 (0.44-7.06) | 1.26 (0.42-6.69) | 1.70 (0.39-7.46) |
| ***Main reason to come to Sweden**  **(ref: As asylum seeker/refugee)** | 1.00 (reference) | | | | | |
| *To work/study |  | 5.17 (1.58-16.89)** |  |  | 4.20 (0.71-24.78) | 2.15 (0.67-6.96) |
| To live with family |  | 2.63 (0.77-8.96) |  |  | 3.16(0.76-13.05) | 1.12 (0.27-4.54) |
| Other |  | (empty) |  |  | (empty) | 1.19 (0.13-11.28) |
| **Having a residence permit**  **(ref: I am an EU/EEA/Swedish citizen)** | 1.00 (reference) | | | | | |
| No |  |  | 2.03 (0.55-7.53) |  | 3.16 (0.61-16.33) | 2.11 (0.13-32.55) |
| Yes |  |  | (omitted) |  | (omitted) | 1.72 (0.10-27.74) |
| **Current living arrangements**  **(ref: with other family)** | 1.00 (reference) | | | | | |
| Alone |  |  |  | 0.92 (0.36-2.32) | 1.84 (0.55-6.11) | 1.07 (0.43-2.66) |
| With friends I knew from earlier |  |  |  | 0.32 (0.07-1.47) | 0.54 (0.08-3.49) | 0.31 (0.05-1.91) |
| In a refugee home |  |  |  | (empty) | (empty) | 0.42 (0.05-3.51) |

Legend: * p<0.05; ** p<0.01

*MENA countries (Algeria, Bahrain, Egypt, Iran, Iraq, Israel, Jordan, Kuwait, Lebanon, Libya, Morocco, Oman, Qatar, Saudi Arabia, Palestine, Syria (excluded), Tunisia, United Arab Emirates, Yemen)

Crude OR for considered explanatory factors. Model 1, Adj. OR for sociodemographic factors: sex, age, country born or raised, religion, number of years living in Sweden, education. Model 2, Adj., OR factors included in Model 1 + Main reason to come to Sweden; Model 3 included Model 1+ Having a residence permit in Sweden; Model 4 included Model 1+Current living arrangements. Model 5 included all variables. Model 6 is multiple imputation
